# The Asylums Board Report

**Published:** 1911-08-26

**Authors:** 


### The Hospital

A Journal of

The Medical Sciences and Hospital Administration.

New Series. No. 239, Vol. IX. [No. 1311, Vol. L.] SATURDAY, AUGUST 26, 1911

### THE ASYLUMS BOARD REPORT.

The annual report of the Metropolitan Asylums Board always contains a large amount of material which is of both medical and institutional interest, and, indeed, of great public importance, too. At the present time, with new experiments in social legislation being tried and impending, it is particularly desirable that their effects shall be closely watched and reported on as soon as possible. In several ways the Asylums Board returns afford information of this sort, and its classification and arrangement by the capable staff of the Board makes it readily available for study. Thus the present report shows clearly how inadvisable it would be at the moment to initiate legislation on the Poor Law. As everyone knows, the Poor Law Commission reported two years ago, and the interval has been filled with the outpourings of those who would reform the Poor Law on lines which it is quite sincerely believed will regenerate our social system. Unfortunately, divergences of opinion among the members of the Commission and among those who have since discussed its report, have been so many and so serious that it is hard for the ordinary layman to know who is right and who is not. Meanwhile another scheme has been put forward by the Association of County Councils as a compromise between conflicting views, but it has scarcely retained on closer examination the approval with which it was at first received. The truth is, as is well remarked in the present Asylums Board report, that the problems to whose solution the Commissioners applied themselves with so much diligence, have changed even since the time when the Commission was sitting, and are further changing every year. Moreover it must of necessity be some time before the effect on Poor Law work of contemporary social legislation can be accurately gauged. Old-age pensions, labour exchanges, steps for dealing with the juvenile labour question, and the Insurance scheme, if it is carried through in its present form—all these must react on the work of the Poor Law authorities to an extent which it is not possible now to forecast; but it cannot be doubted that they will eventually restrict the operation of the Poor Law chiefly to dealings with children and the unemployable, including in the latter term imbeciles and lunatics. There is, therefore, every reason for refraining at present from experimental Poor Law legislation, and especially from such drastic upheavals as those suggested by the much-discussed Minority Report of the Poor Law Commission. A policy of marking time is, in fact, one very much to be advocated just at present, however much it may displease the extremists on either side.

Another very noteworthy happening of the past year was the report sent in by the Board to the Local Government Board on the subject of hospital accommodation. The question had been raised as to the possibility of utilising vacant fever hospitals for the reception of additional classes of sick or defective persons. It transpired that the total available accommodation in London is 9,100 beds for fevers, including small-pox, and 10,700 for children and imbeciles. The senior medical officer of the Local Government Board, Sir Arthur Downes, himself reported that this accommodation is not in excess of what is likely to be required in epidemic times. But at ordinary times many of the fever beds are empty, and the alternatives are either to close them for economy, which involves discharging a trained staff whom it might be difficult to reassemble; or to utilise them for other purposes. The latter was the alternative selected, and as a result of consideration it was decided to devote the Park Hospital, Hither Green, as an additional institution for convalescent children; to convert the Joyce Green Hospital from a small-pox to a general fever hospital; and to arrange for the admission of measles and whooping-cough cases into the fever hospitals as far as the accommodation would allow. A further proposal to admit puerperal fever patients was also favourably reported on by the Asylums Board, but to this the assent of the Local Government Board has not yet been given; also its assent to the reception of measles and whooping cough was limited to pauper cases.

The chapters containing the report of the Finance Committee are also full of interest for hospital managers. It is satisfactory to know that debt is being steadily reduced, and that in eleven years it will be redeemed altogether if no further burdens are laid upon the Board. On fire insurance a report is submitted which may influence other fever hospital authorities up and down the country. Feeling itself unfairly burdened by the insurance companies, the Board has analysed its insurance policies and finds that in twenty years its losses by fire have been trivial compared with the premiums paid. It is believed that the risks in connection with the premises are much less hazardous than the ordinary risks undertaken by the companies; and the Asylums Board has now decided not to insure against fire except in special cases involving ordinary risks. It is quite easy to see that in respect of fever hospitals and lunatic asylums the fire risks must be very small. Thus the buildings are well isolated from each other and from neighbouring premises; they are of fireproof construction, with little or no decorative work of an inflammable nature; the daily life is exceptionally regular, and the staff is continuously on duty day and night; and, finally, fire drill and training are carefully practised and hydrants are liberally supplied. But the policy of non-insurance altogether seems a somewhat dangerous one, and it would surely be preferable to form an insurance fund, similar to that of the great steamship companies, for example. By this means violent fluctuations in the demands made upon the rates would be avoided, and at the same time the ratepayers would still reap the advantage of saving the insurance companies' profits. It is in this connection noteworthy that the managers do not insure their liabilities under the Workmen's Compensation Acts, but meet all claims as they arise themselves. Up to the present this policy has been financially advantageous, but until a longer experience is available the subject will be kept under close review.

